# Institutional Needs

**Published:** 1913-07-05

**Authors:** 


### Institutional Needs.

### A PORTABLE WATER CLOSET.

(Tom Jones's Patent Portable Water Closet or Night Commode, Parkdale, Sedgley, Staffs.)

There can be few who have not experienced the difficulties of finding a night commode for sick-room purposes which has no disadvantages and is at the same time reasonable in price. This new invention is the result of most painstaking consideration of every factor in the problems of design and construction which the ideal commode should solve, and will without doubt prove a great boon to those in whose households such an appliance is found necessary. It is claimed that the Jones's portable commode is not only cheaper than the familiar patterns hitherto employed, but also cleaner, handier, and more sanitary. Moreover, it is free from obnoxious smells when being used, and from obnoxious sights when being emptied. It can, in fact, be carried out of the sick-room and emptied without the person emptying it having any need to see any of the contents; and if water be kept in the bottom the container need never get soiled. The Patent Closet is the same height as an ordinary seat, takes up less room, and is entirely free from risk of breakage or accident to the user, be he ever so clumsy or enfeebled by illness. The price, with non-corrosive receptacle, mahogany lid, and brass hinges, is one guinea.

